# Autism Symptoms in Children and Young Adults With Fragile X Syndrome, Angelman Syndrome, Tuberous Sclerosis Complex, and Neurofibromatosis Type 1: A Cross-Syndrome Comparison

**DOI:** 10.3389/fpsyt.2022.852208

**Published:** 2022-05-16

**Authors:** Kyra Lubbers, Eefje M. Stijl, Bram Dierckx, Doesjka A. Hagenaar, Leontine W. ten Hoopen, Jeroen S. Legerstee, Pieter F. A. de Nijs, André B. Rietman, Kirstin Greaves-Lord, Manon H. J. Hillegers, Gwendolyn C. Dieleman, Sabine E. Mous, ENCORE Expertise Center, Rianne Oostenbrink

**Affiliations:** ^1^ENCORE Expertise Centre for Neurodevelopmental Disorders, Erasmus University Medical Center, Rotterdam, Netherlands; ^2^Department of Child- and Adolescent Psychiatry and Psychology, Erasmus University Medical Center, Rotterdam, Netherlands; ^3^Child Brain Center, Erasmus University Medical Center, Rotterdam, Netherlands; ^4^Department of General Paediatrics, Erasmus MC, Rotterdam, Netherlands; ^5^Clinical Psychology and Experimental Psychopathology Unit, Department of Psychology, Rijksuniversiteit Groningen, Groningen, Netherlands; ^6^Yulius Mental Health, Dordrecht, Netherlands; ^7^Jonx Autism Team Northern-Netherlands, Lentis Mental Health, Groningen, Netherlands

**Keywords:** Fragile X Syndrome, Angelman Syndrome, Tuberous Sclerosis Complex, Neurofibromatosis Type 1, autism spectrum disorder, autistic traits

## Abstract

**Objective:**

The etiology of autism spectrum disorder (ASD) remains unclear, due to genetic heterogeneity and heterogeneity in symptoms across individuals. This study compares ASD symptomatology between monogenetic syndromes with a high ASD prevalence, in order to reveal syndrome specific vulnerabilities and to clarify how genetic variations affect ASD symptom presentation.

**Methods:**

We assessed ASD symptom severity in children and young adults (aged 0-28 years) with Fragile X Syndrome (FXS, *n* = 60), Angelman Syndrome (AS, *n* = 91), Neurofibromatosis Type 1 (NF1, *n* = 279) and Tuberous Sclerosis Complex (TSC, *n* = 110), using the Autism Diagnostic Observation Schedule and Social Responsiveness Scale. Assessments were part of routine clinical care at the ENCORE expertise center in Rotterdam, the Netherlands. First, we compared the syndrome groups on the ASD classification prevalence and ASD severity scores. Then, we compared individuals in our syndrome groups with an ASD classification to a non-syndromic ASD group (nsASD, *n* = 335), on both ASD severity scores and ASD symptom profiles. Severity scores were compared using MANCOVAs with IQ and gender as covariates.

**Results:**

Overall, ASD severity scores were highest for the FXS group and lowest for the NF1 group. Compared to nsASD, individuals with an ASD classification in our syndrome groups showed less problems on the instruments' social domains. We found a relative strength in the AS group on the social cognition, communication and motivation domains and a relative challenge in creativity; a relative strength of the NF1 group on the restricted interests and repetitive behavior scale; and a relative challenge in the FXS and TSC groups on the restricted interests and repetitive behavior domain.

**Conclusion:**

The syndrome-specific strengths and challenges we found provide a frame of reference to evaluate an individual's symptoms relative to the larger syndromic population and to guide treatment decisions. Our findings support the need for personalized care and a dimensional, symptom-based diagnostic approach, in contrast to a dichotomous ASD diagnosis used as a prerequisite for access to healthcare services. Similarities in ASD symptom profiles between AS and FXS, and between NF1 and TSC may reflect similarities in their neurobiology. Deep phenotyping studies are required to link neurobiological markers to ASD symptomatology.

## Introduction

Autism spectrum disorder (ASD) is a heterogeneous neurodevelopmental disorder defined by impairments in social communication, restricted or repetitive behaviors or interests, and hyper- or hyposensitivity to sensory stimuli. ASD occurs in around 1.7% of the general population (1). Despite the rapid discovery of genes related to ASD, and the high heritability estimates (64–91%), the exact etiology of ASD remains unclear (2, 3). Studying ASD symptoms in genetically homogenous groups could clarify the pathway from genes to behavior. Genetic syndromes with high ASD prevalence rates include Fragile X Syndrome (FXS), Angelman syndrome (AS), Tuberous Sclerosis Complex (TSC) and Neurofibromatosis type 1 (NF1) (4). Despite their unique genetic variation, these syndromes show similarities in their neurodevelopmental pathways. These syndromes are all affected by alterations in the mechanistic target of rapamycin (mTOR) pathway (5–10), which has also been related to non-syndromic ASD (nsASD) (10, 11). FXS and AS are similar in that both syndromes show atypical DNA methylation that results in increased levels of Activity-Regulated Cytoskeleton-associated protein (Arc), causing reduced synaptic plasticity and disruptions in cerebral development which often lead to intellectual disability (8). TSC and NF1 are similar in that they are both affected by genetic variations that inactivate tumor-suppressor genes. This inactivation leads to an over-activation of mTOR, which increases the risk of tumors in the nervous system that may affect brain development and function (7, 8). In these syndromes, a unique spectrum of ASD symptoms seems to be present (12–17). If differences or similarities in the affected pathways are also reflected by differences or similarities in ASD symptom presentation, this might help identify factors that contribute to the development of specific ASD symptoms, or the development of ASD in general. As a step toward linking ASD symptoms to specific neurobiological pathways, several studies have been conducted to describe ASD symptomatology in monogenetic neurodevelopmental disorders in detail.

FXS affects ~1 in 4,000 males and 1 in 8,000 females (18, 19), and is one of the leading inherited causes of autism and developmental delay (4). FXS is caused by an CGG repeat expansion in the Fragile X Mental Retardation 1 (*FMR1*) gene, which leads to reduced synaptic plasticity, complications with dendritic development and problems with neurogenesis (20–24). Due to the X-linked nature of FXS, males are generally more severely affected than females (males: 20 < IQ < 70 (25), females: 70 < IQ < 90) (26, 27). It is estimated that about 15 to 36% of people with a full Fragile X Syndrome mutation meet the clinical criteria for an assessment-based ASD classification (4, 28–30). Even without meeting all the criteria of ASD, the majority of people with FXS express behavior related to autism (30–33). Studies have shown that, compared to nsASD, individuals with FXS and ASD show less impairment on social and communication domains (32–35), more social anxiety (16), more problems with restricted and repetitive behavior, and less compulsive and ritualistic behavior (32–35).

Angelman Syndrome (AS) can be caused by different genetic variations that affect the expression of the *UBE3A* gene in the chromosomal region 15q11-q13 (36). AS is characterized by cognitive impairments, lack of speech, motor dysfunction, and epilepsy (37). The intellectual development of individuals with AS usually does not exceed a mental age of 24 to 30 months, regardless of their chronological age (38, 39). The prevalence of assessment-based ASD classifications in AS ranges from 20 to 80% in the literature (4, 40–42). While several studies have found that genetic variation within chromosomal region 15q11-q13 is independently associated with ASD (43), a meta-analysis revealed no such relation. Therefore, the precise effect of *UBE3A* variations on the development of ASD remains unclear (44). Compared to people with nsASD, people with AS and ASD display significantly less impairment in areas such as social smile, facial expressions directed to others, shared enjoyment in interaction, response to name and unusual interests or repetitive behavior (40). Compared to FXS, individuals with AS appear to be more sociable (12), and while both syndromes have altered sensory processing, their response to sensory stimuli is not similar (13).

TSC is caused by genetic variations in either the *TSC1* or *TSC2* gene, leading to mTOR overstimulation (45). This activation induces cellular and tissue dysplasia, causing tumorigenesis that can affect multiple organs (45, 46). Since the central nervous system is almost always afflicted, epilepsy and neuropsychiatric disorders are often seen (46, 47). While approximately half of individuals with TSC score within the normal range of cognitive ability, the other half shows mild to severe (14.5%, 25 ≤ IQ < 70) or profound (30.5%, IQ < 25) intellectual disability (48). The estimated prevalence of assessment-based ASD classifications in TSC ranges from 35 to 60% (4, 17, 47, 49, 50), of which the severity may be influenced by presence of epilepsy (49). Jeste et al. showed that impairments in social communication in children with TSC do not differ from those in children with nsASD (17). Recently, molecular target therapy with mTOR inhibitors has demonstrated a reduction of epilepsy symptoms in people with TSC (51, 52), and the reduction of autism symptoms in animal models (53, 54).

In NF1, a genetic variation in the *NF1* gene causes a neurofibromin deficiency, which inhibits the cell cycle and cell differentiation, and enables unrestricted cell growth (55). Tumor growth in NF1 can cause various neurologic, cardiovascular, gastrointestinal, endocrinal and orthopedic complications (56–60). However, the most common challenge for children with NF1 are learning and behavioral problems (61–63). The average IQ of individuals with NF1 lies around 90, with 6 to 7% having an IQ lower than 70 (15, 64). The prevalence of assessment-based ASD classifications in NF1 ranges from 10 to 39% (4, 15, 49, 65). Some studies have found that NF1 has a unique ASD phenotype with better eye contact, less repetitive behavior and more severe autistic mannerisms compared to individuals with nsASD (66, 67). However, others have shown that NF1 shows a symptom profile similar to that in both nsASD and TSC (49).

In summary, FXS, TSC, AS and NF1 have a high prevalence of ASD symptoms and are caused by unique and well-described genetic variations, making them ideal candidates to study genotype-phenotype relationships in ASD. A direct comparison of these syndromes, in which ASD symptoms are assessed in the same clinical setting and with the same diagnostic instruments, has not yet been done. The main aim of this large cohort study is to identify differences and similarities in ASD symptom severity between these monogenetic developmental disorders, as well as compared to a non-syndromic ASD group. Based on earlier studies, we expect the FXS group to display the most severe symptoms, especially on the domains of stereotypic behavior and limited interests, while children with TSC and NF1 will show less severity on these domains. In contrast, we expect less severe symptoms for the FXS and AS groups in social interaction. Based on the similarities in the neurobiological pathways between FXS and AS, and between NF1 and TSC, respectively, we will explore if and how these similarities may be reflected in the ASD symptom profiles of these groups.

Besides linking genetic pathways to behavior, the ASD symptom profiles in these syndrome groups could help us understand and value the information gathered with diagnostic instruments that are developed using non-syndromic norm groups. This may add to the discussion of whether a categorical approach in ASD diagnostics is appropriate in syndromic ASD as well as non-syndromic ASD, or whether a symptom-based approach is more suitable (68). A forced fit between symptom presentation and scoring procedures may result in a loss of clinically important information or the under- or over diagnosis of ASD within these groups, which may directly impact clinical decision making and an individual's access to health services (33). Additionally, as treatment of ASD symptoms requires a personalized stepped-care approach (69), the syndrome specific symptom profiles might reveal syndrome-specific targets for treatment and intervention.

## Methods

### Participants

**Figure 1:**
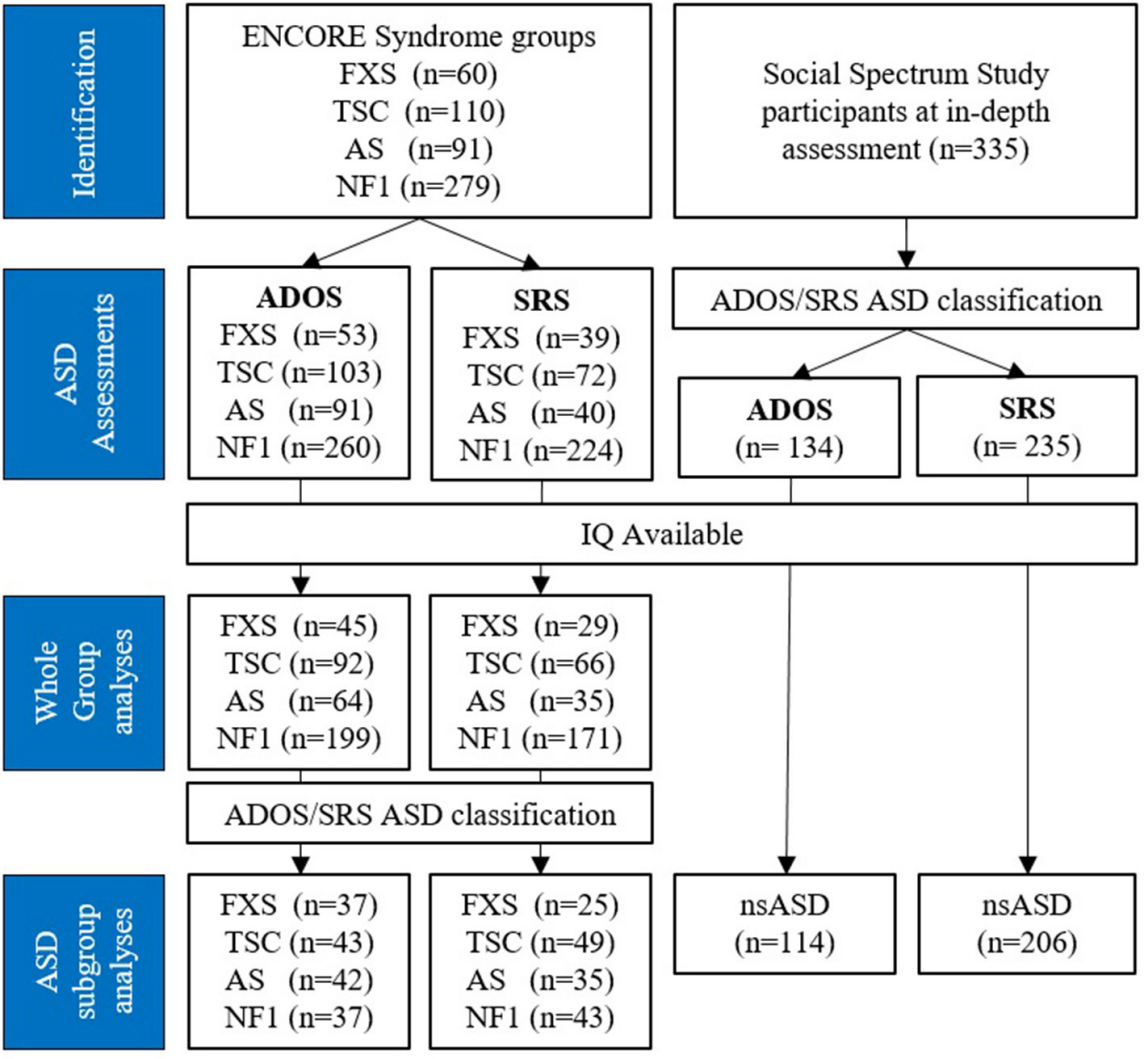
Flow chart of inclusion process. ASD, Autism Spectrum Disorder; FXS, Fragile X Syndrome; TSC, Tuberous Sclerosis Complex; AS, Angelman Syndrome; NF1, Neurofibromatosis type 1; nsASD, non-syndromic ASD; ADOS, Autism Diagnostic Observation Schedule; SRS, Social Responsiveness Scale.

We included four groups of children and young adults (aged 0–28 years) with syndromes that have high ASD prevalence: FXS, AS, NF1, and TSC, see Figure 1. ASD symptoms and cognitive functioning were assessed as part of routine clinical care that is performed in all children seen at the ENCORE expertise center for genetic neurocognitive developmental disorders within the Erasmus MC Sophia Children's Hospital in Rotterdam, the Netherlands. All children with these syndromes were included, regardless of their ASD symptomatology.

In addition, we included an nsASD group as a frame of reference. For this group, we used data collected as part of the Social Spectrum Study, a clinical cohort study that aimed to identify children at risk for ASD by screening children who had been referred to six large child and adolescent mental health services in the South-West of the Netherlands. A detailed description of the sample characteristics and data collection procedure of this group can be found in the study design paper of the Social Spectrum Study (70). We included children that participated in the in-depth assessment (T1), which consisted of all children with an ASD classification on the Social Responsiveness Scale (SRS) (71) at initial screening (T0) (*N* = 235) and a random selection of children that screened negative at T0 (*N* = 100). From this group, we included the children with an ASD classification according to the Autism Diagnostic Observation Schedule (ADOS) (72) and/or the SRS in our subgroup analyses. None of these children were diagnosed with a genetic syndrome.

### Measures

#### Autism Spectrum Symptoms

To assess ASD symptom severity, two diagnostic instruments were used: the ADOS and the SRS. Both instruments provide valuable insight into a child's behavior and are therefore often used together in clinical practice, which is also the case for children seen at ENCORE.

##### ADOS

The ADOS uses a semi-structured schedule of activities to allow researchers or clinicians to observe an individual's behavior in areas associated with a diagnosis of ASD: social communication, reciprocal social interactions and restricted and repetitive behavior. To suit an individual's developmental level and language level, one of five modules is selected. Items vary across the modules. The sensitivity of the ADOS-2 is between 72 and 97% and the specificity is between 19 and 94%, depending on the module used (73).

Data was collected by certified clinicians, using the ADOS-G (3.6%) (72) and ADOS-2 (96.4%) (74). ADOS-G scores were converted to ADOS-2 scores *via* the manual (74). Calibrated severity scores (CSS) are computed for the total score, and for the domains Social Affect (SA) and Restricted and Repetitive Behavior (RRB). CSS correct for chronological-age and range between 1 and 10, with higher scores indicating more severe ASD symptoms. For children younger or older than the available norm groups we calculated scores based on the nearest available norm group. Based on the Total CSS, individuals classify as “non-spectrum” (scores 1–3), “ASD” (scores 4–5) or “Autism” (scores >5) (75, 76). A Total CSS > 3 was used as a cut-off for the ASD classification used in our analyses. It is important to note however, that the RRB CSS, scored 1 to 10, are converted from the raw RRB subscale scores that range from 0 to 7. The raw scores are converted to a 1–10 scale by making scores 2, 3, and 4 impossible to obtain. While this improves the ease of interpretation relative to the other CSS scores, it is problematic for data analysis (14). Therefore, we used the original CSS-RRB scores on a 0–7 scale in our analyses.

The ADOS groups items into five subscales (language and communication, reciprocate social interaction, creativity/play, restricted and repetitive behavior, and other behavior), but does not provide normed subscale scores. For our ASD profile analyses, we computed a “Weighted Subscale Score” (WSS) for each subscale that accounts for the differences in the number of items between both the subscales and the ADOS modules. The WSS were calculated by dividing the individual's total score per subscale through the subscale's maximum score, resulting in a score between 0 and 1, with higher scores representing higher autism severity. Because Subscale E includes 3 items that measure different constructs, we did not calculate a weighted score for this subscale.

##### SRS

In this 65-item ASD screening questionnaire, parents or caregivers rate their child's behavior over the past 6 months on a 4-point Likert scale, with higher scores indicating higher symptom severity. Each item belongs to one of five subscales: Social Awareness, Social Cognition, Social Communication, Social Motivation, and Autistic Mannerisms. Age- and gender-normed T-scores can be computed for the total score (SRS_TOT_) and for the domains “Social Communication and Interaction” (SRS_SCI_, consisting of the first four subscales) and “Restricted Interests and Repetitive Behavior” (SRS_RRB_, consisting of the Autistic Mannerisms subscale). In addition, T-scores can be calculated for all subscales. Based on the T-scores, ASD symptom severity is interpreted as non-clinical (T < 60), mild (60 ≤ T ≥ 75) or severe (T > 75). An SRS_TOT_ T-score > = 60 was used as a cut-off for the ASD classification used in our analyses. The sensitivity of the SRS-2 is 93%, with a specificity of 91% (38). Our sample included data from both the SRS and SRS-2. As items do not differ between the SRS and the SRS-2, all cases were classified using the SRS-2 classification methods, regardless of the questionnaire used. For children younger or older than the available norm groups we calculated scores based on the nearest available norm group.

#### Cognitive Functioning

Children with developmental delay are more likely to score in the clinical range of both the ADOS (77) and SRS (78). As developmental levels differ between the syndrome groups, we included intellectual functioning as a covariate in the group comparisons. Cognitive functioning was assessed using an age- or developmental level appropriate instrument. These instruments include the Wechsler preschool and primary scale of intelligence (WPPSI-III-NL) (79), the Wechsler intelligence scale for children [WISC-III-NL (80) or WISC-V-NL (81)], the Wechsler Adult Intelligence Scale (WAIS-III) (82), the Wechsler Non Verbal scale of Ability (WNV) (83), the Bayley Scales of Infant and Toddler Development third edition (Bayley-III) (84) and the Snijders-Oomen Non-verbal intelligence test (SON) (85). In some cases, the chronological age of the individual was higher than the available norm groups (e.g., when an 18-year-old with developmental delay was assessed using the WISC). In these cases, no full-scale IQ could be calculated and a developmental quotient (DQ) was computed instead (DQ = estimated developmental age/chronological age × 100, with M = 100, SD = 15).

### Procedure

For the syndrome groups we included individuals with a complete ADOS and/or SRS assessment between May 2009 to March 2021. In case complete data from multiple time points was available for an individual, we selected the most recent time point. This resulted in 507 cases for the ADOS and 375 cases for the SRS. The nsASD group data consisted of 134 ADOS assessments and 235 SRS questionnaires, collected between May 2011 and December 2013.

### Statistical Analysis

As each instrument has a unique focus, separate analyses were performed for the ADOS and the SRS data. First, we compared the syndrome groups on the prevalence of ADOS and SRS ASD classifications using Chi square tests. Next, we compared the main ASD severity scores (ADOS: CSS_TOT_, CSS_SA_, and CSS_RRB_; SRS: T_TOT_, T_SCI_, T_RRB_) between the syndrome groups using MANCOVA's with developmental level (IQ/DQ) and gender as covariates. We first did this for the syndrome groups as a whole. Then, we compared the syndrome groups again while only including individuals with an ASD classification on the ADOS or SRS, respectively, in order to include all individuals with a clinical score on that particular instrument irrespective of their classification status on the other instrument, with the nsASD group added as reference. We did this to reduce the expected within-group variability in ASD severity. Finally, for our ASD profile analysis, we also compared the ASD subgroups on subscales of the ADOS and SRS.

Data analyses were performed in IBM SPSS Statistics version 25 (86). We use an alpha level of 0.05 for all analyses and applied a Bonferroni correction for multiple comparisons in our *post-hoc* comparisons. In all tables the uncorrected-mean-scores, standard deviations, F-statistics, Bonferroni-corrected-*p*-values and the effect sizes (partial η^2^) are provided. Without a suitable alternative, MANCOVA's were used despite the skewed data (see Supplementary Figure 1) and correlation between the covariates and the predictor, so our results should be interpreted cautiously.

### Missing Data

A non-response analysis for missing IQ/DQ scores in the syndrome groups revealed no differences in ADOS or SRS ASD severity scores between individuals with and without available IQ/DQ scores. For the nsASD group however, severity scores were higher for those without an available IQ/DQ score than for those with an IQ/DQ score for the ADOS [*t*_(266)_ = −2.087, *p* = 0.038]. Because we expected the effect of IQ/DQ to be substantial in the syndrome groups we included IQ as a covariate nonetheless.

## Results

### Descriptives

Sample characteristics are presented in Table 1. We found significant differences in ASD classification prevalence between the syndrome groups for both the ADOS and the SRS. According to both instruments, approximately a quarter of individuals in the NF1 group received an ASD classification, which was lower compared to the FXS, TSC, and AS groups in which at least half of individuals received an ASD classification. The ASD classification prevalence was highest in the FXS and AS groups. An overview of ASD classifications in the syndrome groups is provided in Supplementary Figure 2.

**Table 1:** Sample characteristics.

	**FXS**	**TSC**	**AS**	**NF1**	**nsASD**	**Cross-syndrome comparison**		
						***df***	***X***	***p***
**ADOS (*****N*** **=** **641)**								
*N*	53	103	91	260	134			
Age M (SD)	9.00 (5.32)	9.54 (4.92)	8.85 (5.05)	7.22 (3.45)	6.82 (2.34)			
Age range (y)	2–28	2–19	2–21	1–18	2–12			
Males *N* (%)	41 (77.4)	54 (52.4)	47 (51.6)	143 (55.0)	113 (84.3)			
**IQ/DQ**								
*N* (%)	45 (84.9)	92 (89.3)	64 (70.3)	199 (76.5)	114			
M (SD)	49.6 (19.1)	63.9 (29.6)	19.0 (10.7)	87.0 (15.7)	92.2 (17.7)			
range	20–93	4–127	3–52	38–135	50–141			
ASD class *N* (%)	44 (83.0)_a_	50 (48.5)_b_	60 (65.9)_ab_	52 (20.0)_c_	–	3	112	<0.001
**SRS (*****N*** **=** **635)**								
*N*	39	72	40	224	235			
Age M (SD)	8.10 (5.50)	9.68 (5.16)	9.20 (4.70)	7.02 (3.34)	7.17 (1.97)			
Age range (y; m)	0;9–26	1–19	2–20	1–17	4–11			
Males *N* (%)	29 (74.4)	33 (45.8)	22 (55.0)	121 (54.0)	195 (75.0)			
**IQ/DQ**								
*N* (%)	29 (74.4)	66 (91.7)	35 (87.5)	171 (76.3)	206			
M (SD)	53.4 (20.9)	64.7 (26.3)	16.8 (10.6)	88.4 (15.7)	95.9 (16.7)			
range	22–93	7–127	3–52	38–135	50–145			
ASD class *N* (%)	35 (89.7)_ab_	54 (75.0)_b_	38 (95.0)_a_	65 (29.0)_c_	–	3	114	<0.001

*For syndrome groups with the same subscript letter the prevalence of an ASD classification is not significantly different at the p = 0.05 level. ASD, Autism Spectrum Disorder; FXS, Fragile X Syndrome; TSC, Tuberous Sclerosis Complex; AS, Angelman Syndrome; NF1, Neurofibromatosis Type 1; nsASD, non-syndromic ASD; DQ, Developmental Quotient; y, years; m, months*.

### Cross-Syndrome Comparisons of Symptom Severity Scores

#### ADOS

**Figure 2:**
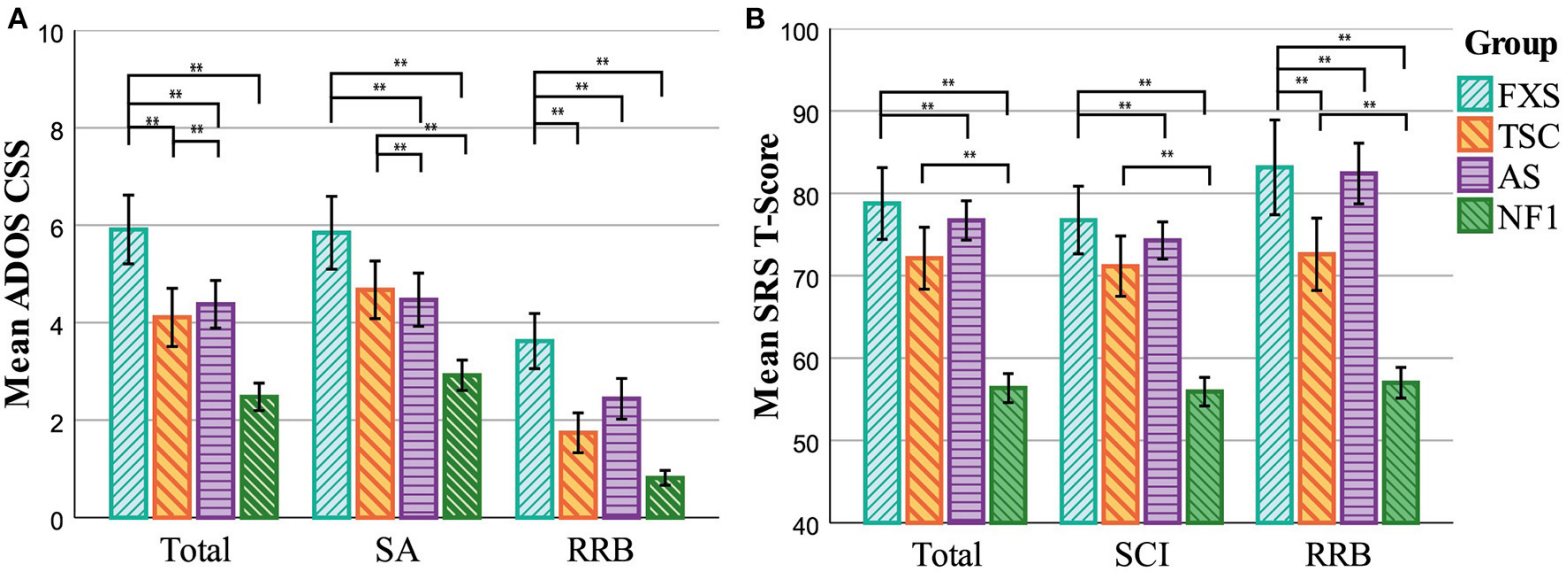
ASD severity scores per syndrome group. **(A)** Means represent the ADOS scores of the whole group, regardless of the presence of an ADOS ASD classification. **(B)** Means represent the SRS T-scores of the whole group, regardless of the presence of an SRS ASD classification. FXS, Fragile X Syndrome; TSC, Tuberous Sclerosis Complex; AS, Angelman Syndrome; NF1, Neurofibromatosis Type 1; CSS, Calibrated Severity Score; SA, Social Affect; RRB, Restricted (Interests) and Repetitive Behavior; SCI, Social Communication and Interaction. The bars represent the uncorrected mean scores of the groups. Significant group differences of the MANCOVA *post hoc* comparisons are presented. Error bars represent 95% CI, **p < 0.01, Bonferroni corrected.

ASD severity scores of the whole-group cross-syndrome comparison are presented in Figure 2A and Table 2. We found a significant main effect of group for the ADOS CSS scores collectively and individually. *Post hoc* pairwise comparisons revealed that for the total CSS, autism severity was higher in the FXS group compared to all other groups, and higher in the TSC group compared to the AS group. For the social affect domain, the FXS and TSC groups had higher severity scores than the AS and NF1 groups. Lastly, the FXS group had higher severity scores on the restricted and repetitive behavior domain compared to all other groups.

**Table 2:** Cross-syndrome comparisons of symptom severity scores.

**Dependent variable**	***df***	***df* error**	**F**	**η^2^**	**FXS**	**TSC**	**AS**	**NF1**
					**M (SD)**	**M (SD)**	**M (SD)**	**M (SD)**
**ADOS CSS—Whole sample**								
Multivariate	9	954	9.238^**^	0.066	*Wilks' Λ = 0.816*			
CSS_TOT_	3	394	15.877^**^	0.108	5.91 (2.34)_a_	4.11 (2.89)_b_	4.38 (1.96)_c_	2.48 (2.02)_bc_
CSS_SA_	3	394	12.654^**^	0.088	5.84 (2.34)_a_	4.67 (2.85)_a_	4.47 (2.92)_b_	2.92 (2.24)_b_
CSS_RRB_	3	394	19.166^**^	0.127	3.62 (1.89)_a_	1.74 (1.97)_b_	2.44 (1.68)_b_	0.81 (1.79)_b_
**SRS T-scores—Whole sample**								
Multivariate	9	713.2	7.868^**^	0.074	*Wilks' Λ = 0.794*			
T_TOT_	3	295	17.295^**^	0.150	78.8 (15.1)_a_	71.7 (15.2)_ab_	77.5 (6.5)_bc_	55.7 (12.4)_c_
T_SCI_	3	295	16.341^**^	0.142	76.7 (14.3)_a_	70.8 (15.0)_ab_	74.8 (6.3)_bc_	55.2 (12.2)_c_
T_RRB_	3	295	17.300^**^	0.150	83.6 (18.7)_a_	72.0 (17.7)_b_	83.6 (10.5)_bc_	56.2 (13.2)_c_

*The means represent the uncorrected mean scores of the groups. For groups with the same subscript letter the mean CSS or subscale score is not significantly different at the p = 0.05 level (Bonferroni corrected). FXS, Fragile X Syndrome; TSC, Tuberous Sclerosis Complex; AS, Angelman Syndrome; NF1, Neurofibromatosis Type 1; CSS, Calibrated Severity Score; Tot, Total score; SA, Social Affect; RRB, Restricted and Repetitive Behavior; T_TOT_, T-score SRS total score; T_SCI_, T-score Social Communication and Interaction; T_RRB_, T-score Restricted interests and repetitive behavior*.

** *p < 0.01*.

#### SRS

The comparison of SRS severity scores also revealed a significant effect of group for the collective and individual T-scores, see Figure 2B and Table 2. Similar to the results of the ADOS, *post hoc* pairwise comparisons showed that the FXS group had higher severity scores than the NF1 and AS groups for all subscales, and higher severity scores compared to all other groups on the Restricted Interests and Repetitive Behavior domain. While for the ADOS the TSC group had higher ASD severity scores compared to the NF1 group specifically on the Social Affect domain, for the SRS the TSC group had higher severity scores compared to the NF1 group on all severity scores.

### Cross-Syndrome Comparisons of Symptom Severity Scores in Individuals With an ASD Classification

#### ADOS

**Figure 3:**
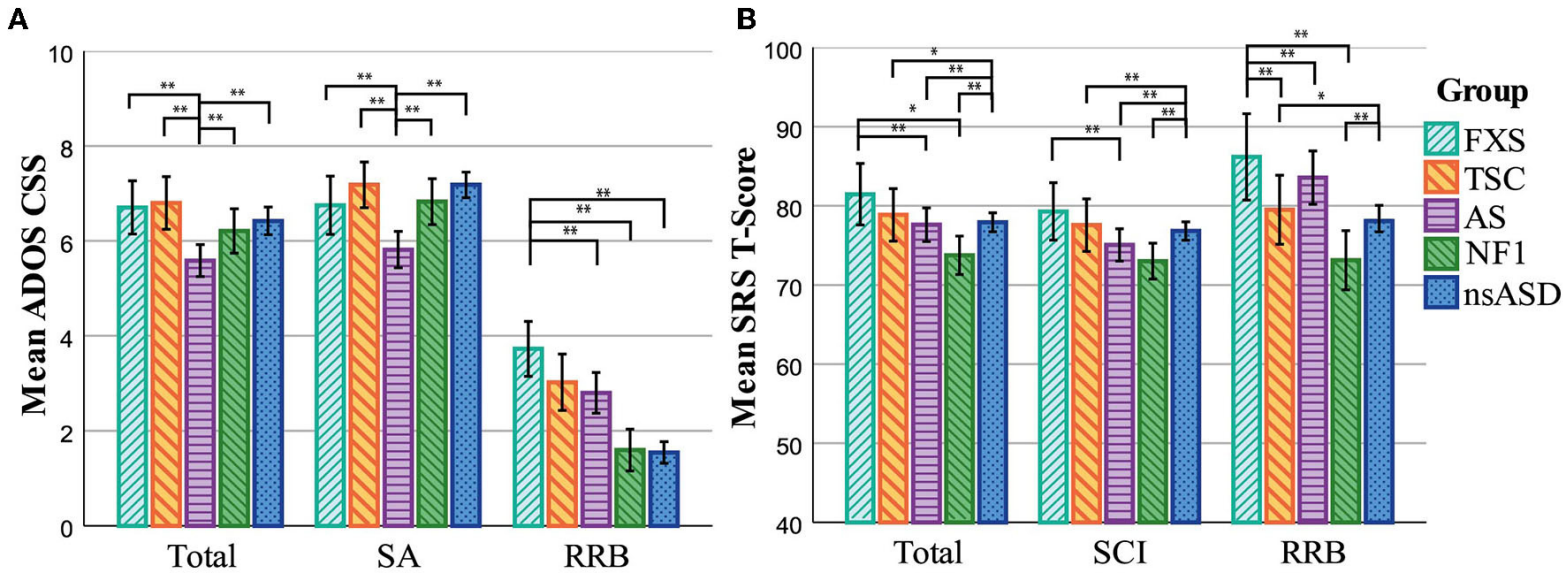
Cross-syndrome comparisons of symptom severity scores in individuals with an ASD classification. **(A)** Means represent the ADOS scores. **(B)** Means represent the SRS T-scores. FXS, Fragile X Syndrome; TSC, Tuberous Sclerosis Complex; AS, Angelman Syndrome; NF1, Neurofibromatosis Type 1; nsASD, non-syndromic ASD; CSS, Calibrated Severity Score; SA, Social Affect; RRB, Restricted (Interests) and Repetitive Behavior; SCI, Social Communication and Interaction. The bars represent the uncorrected mean scores of the groups. Significant group differences of the MANCOVA *post hoc* comparisons are presented. Error bars represent 95% CI, *p < 0.05, **p < 0.01, Bonferroni corrected.

When including only individuals with an ASD classification on the ADOS, a group comparison of the ADOS severity scores again showed a main effect of Group for the CSS scores collectively and individually (see Figure 3A and Table 3). *Post hoc* pairwise comparisons showed that for both the Total and Social Affect CSS, severity scores in the individuals with an ASD classification in the FXS, TSC, and NF1 groups did not differ significantly from the severity scores of the nsASD group. Only individuals with an ADOS ASD classification in the AS group had lower severity scores compared to the nsASD group, as well as compared to all other syndrome groups. For Restricted and Repetitive Behavior, the severity scores were higher for individuals with an ADOS ASD classification in the FXS group compared to the nsASD group, as well as the AS and NF1 groups.

**Table 3:** Cross-syndrome comparisons of symptom severity scores in individuals with an ASD classification.

**DV**	***df***	***df* error**	**F**	**η^2^**	**FXS**	**TSC**	**AS**	**NF1**	**nsASD**
					**M (SD)**	**M (SD)**	**M (SD)**	**M (SD)**	**M (SD)**
**ADOS CSS—ASD subsample**									
Multivariate	12	701.4	4.951^**^	0.069	*Wilks' Λ = 0.860*			
CSS_TOT_	4	267	5.757^**^	0.079	6.68 (1.91)_a_	6.77 (1.35)_a_	5.50 (1.79)_b_	6.11 (1.68)_a_	6.36 (1.69)_a_
CSS_SA_	4	267	5.980^**^	0.082	6.62 (1.79)_a_	7.14 (1.91)_a_	5.71 (1.55)_b_	6.73 (1.79)_a_	7.12 (1.59)_a_
CSS_RRB_	4	267	8.351^**^	0.111	3.89 (1.81)_a_	2.95 (2.13)	2.79 (1.77)_b_	1.57 (1.56)_b_	1.43 (1.16)_b_
**SRS T-scores—ASD subsample**									
Multivariate	12	926.3	3.396^**^	0.037	*Wilks' Λ = 0.892*			
T_TOT_	4	352	8.794^**^	0.091	82.6 (12.5)_ac_	78.1 (11.2)_a_	77.5 (6.5)_b_	73.5 (9.0)_b_	78.4 (9.1)_c_
T_SCOM_	4	352	7.966^**^	0.083	80.2 (11.8)_ac_	76.9 (11.4)_a_	74.8 (6.3)_b_	72.7 (8.7)_a_	77.3 (8.9)_c_
T_RRB_	4	352	6.438^**^	0.068	87.8 (16.0)_a_	78.5 (15.0)_b_	83.6 (10.5)_bc_	73.4 (12.6)_b_	78.7 (12.8)_ac_

*The means represent the uncorrected mean scores of the groups. For groups with the same subscript letter the mean CSS or subscale score is not significantly different at the p = 0.05 level (Bonferroni corrected). No subscale letter means the group does not differ from any other group. DV, Dependent Variable; FXS, Fragile X Syndrome; TSC, Tuberous Sclerosis Complex; AS, Angelman Syndrome; NF1, Neurofibromatosis Type 1; nsASD, non-syndromic ASD; CSS, Calibrated Severity Score; TOT, Total score; SA, Social Affect; RRB, Restricted and Repetitive Behavior; T_TOT_, T-score SRS total score; T_SCI_, T-score Social Communication and Interaction; T_RRB_, T-score Restricted interests and repetitive behavior*.

** *p < 0.01*.

#### SRS

The group comparison of SRS severity scores in individuals with an SRS ASD classification again showed a significant main effect of Group for the collective and individual T-scores (see Table 3 and Figure 3B). While for the ADOS only the AS group severity scores were lower compared to the nsASD group on the total and Social Affect severity scores, the SRS *post hoc* comparisons showed that the nsASD group had higher severity scores compared to individuals with an ASD classification in the TSC, AS and NF1 groups on these two subscales. Also unlike the ADOS, there was no difference in severity scores between individuals with an ASD classification in the FXS group and the nsASD group on the SRS Restricted Interests and Repetitive Behavior domain. The nsASD group did show higher severity scores on the SRS Restricted Interests and Repetitive Behavior domain compared to individuals with an SRS ASD classification in the NF1 and TSC groups.

When we compare the syndrome groups to each other, individuals with an ASD classification in the FXS group had higher severity scores on the total score compared to individuals with an ASD classification in the AS and NF1 groups, higher scores compared to individuals with an ASD classification in the AS group on the Social Communication and Interaction domain, and higher severity scores compared to all other syndrome groups on the Restricted Interests and Repetitive Behavior domain. Unlike for the ADOS, we did not find differences in SRS severity scores between individuals with an ASD classification in the TSC, AS and NF1 groups.

### Cross-Syndrome Symptom Profile Analysis in Individuals With an ASD Classification

#### ADOS

**Figure 4:**
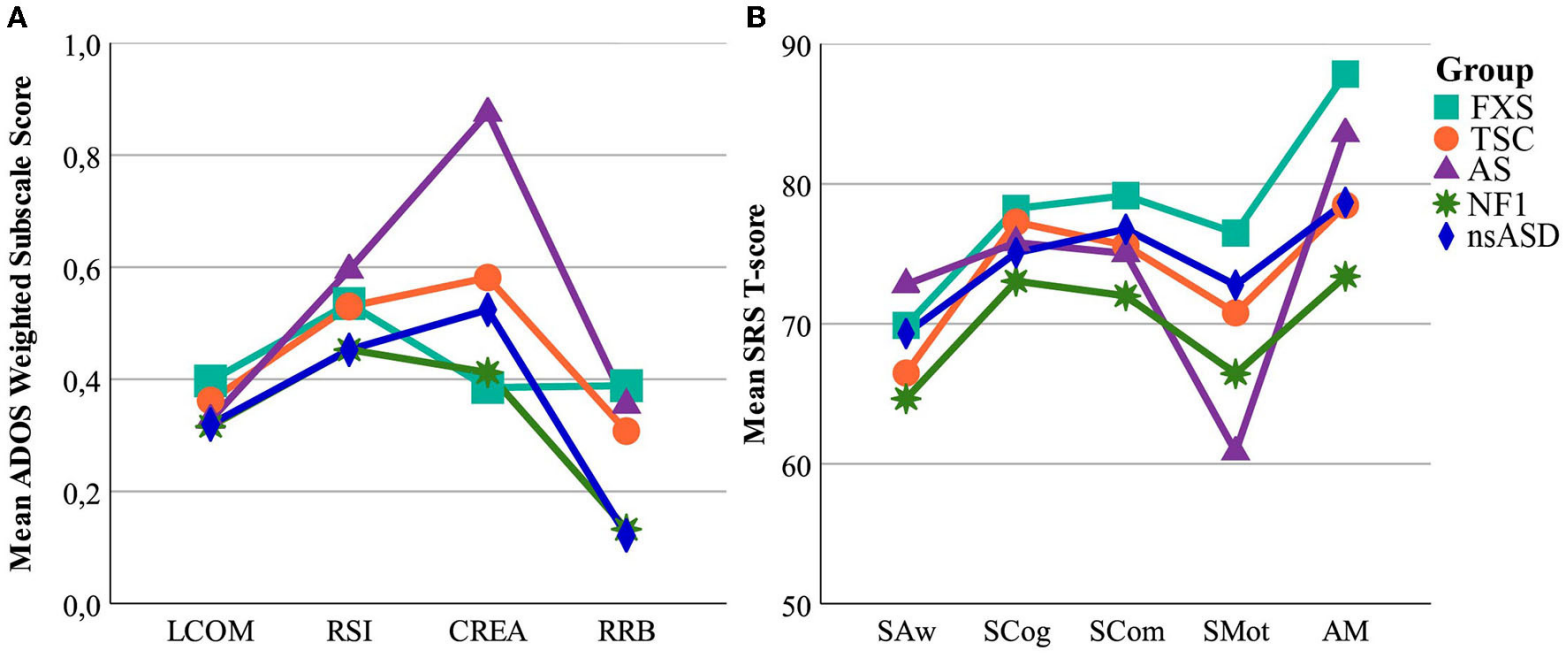
ASD symptom severity profile per syndrome group in the ASD subsample. **(A)** Means represent ADOS Weighted Subscale Scores. **(B)** Means represent SRS subscale T-scores. FXS, Fragile X Syndrome; TSC, Tuberous Sclerosis Complex; AS, Angelman Syndrome; NF1, Neurofibromatosis Type 1; nsASD, non-syndromic Autism Spectrum Disorder; WSS, Weighted subscale score; LCOM, Language and communication; RSI, Reciprocal Social Interaction; CREA, Creativity and Play subscale; RRB, Restricted and Repetitive Behavior; SAw, Social Awareness; SCog, Social Cognition; SCom, Social Communication; SM, Social Motivation; AM, Autistic mannerisms.

The results of the cross-syndrome comparison of ASD symptom profiles are presented in Figure 4 and Supplementary Table 1. Individuals with an ASD classification on the ADOS in the FXS and AS group had similar profiles for all subscales except creativity and play, for which the AS group had higher severity scores than the FXS group, as well as the NF1 group. The profiles of individuals with an ADOS ASD classification in the NF1 and TSC groups were similar to each other, as well as to the profile of the nsASD group, for all subscales.

#### SRS

Contrary to the ADOS, the profiles of individuals with an ASD classification on the SRS in the FXS and AS groups showed little similarity for the SRS, as the FXS group showed higher severity scores compared to the AS group on all subscales except Social Awareness. Instead, the profile of the FXS group was similar to that of the nsASD group on all subscales. The AS group on the other hand showed lower severity scores compared to the nsASD group on the Social Cognition, Social Communication and Social Motivation subscales. Similar to the ADOS, individuals with an ASD classification in the NF1 and TSC groups shared a similar ASD profile for all subscales of the SRS, but their profiles were not similar to that of the nsASD group.

## Discussion

The main aim of this study was to identify differences and similarities in ASD symptomatology between monogenetic syndromes with high ASD prevalence—FXS, TSC, NF1, and AS—that may reveal how different genetic variations affect ASD symptom severity.

### ASD Classification Prevalence

In line with the literature, for both instruments, the ASD classification prevalence was highest in the FXS group and lowest in the NF1 group. The prevalence of ADOS and SRS ASD classifications we found for the FXS group (around 80–90%), and the AS group (70–90%) fell on the high end of the range found in the literature. Overall, ASD classification prevalence seemed to be higher for the SRS compared to the ADOS. This difference was especially high for the TSC (26% difference) and AS groups (29% difference), although we did not test this statistically as the samples contained different individuals. The difference in ASD classification prevalence between the ADOS and SRS is likely the result of the fundamental differences between the instruments. First, as a screening instrument the SRS is required to be highly sensitive by design. Secondly, the caregiver's perspective provided by the SRS may be more subjective. Thirdly, the setting and time period that is covered by these instruments may affect the scores, as some children may have learned to present more socially desirable behavior in unfamiliar surroundings than they would do in the comfort of their own home. Alternatively, they may experience more anxiety in the presence of an unfamiliar individual.

An important factor that may explain the higher prevalence of ADOS and SRS ASD classifications in children with FXS and AS is their developmental level. Generally, individuals with FXS and AS experience more severe intellectual disability or developmental delay compared to NF1. Cognitive impairment is known to affect scores on ASD screening instruments (75), and has been related to reduced specificity of the ADOS, especially for young children (73). Cognitive impairment additionally impacts whether individuals qualify for a DSM-5 classification, which impacts their access to health care services (33). The DSM-5 requires that difficulties in social communication must be lower than could be expected based on the developmental level of the individual, so that the deficiencies in social skills cannot be attributed to developmental delay. Therefore, it is likely that difficulties in social communication in individuals with FXS and AS are more frequently attributed to developmental delay rather than to the presence of ASD. This means that children with intellectual disability or developmental delay can score in the clinical range on screening or diagnostic instruments but not qualify for a formal DSM-5 ASD classification, even though they may experience the same symptom severity. At the same time, children with intellectual disability or developmental delay who qualify for a formal DSM-5 ASD classification are likely to show more severe symptoms compared to children with a formal DSM-5 ASD classification without intellectual disability or developmental delay. Even though we controlled for IQ/DQ in our analyses, it remains unclear whether the symptoms we see in our sample are beyond their expected developmental level.

Given the wide variety of symptom profiles that exist, in both syndromic and non-syndromic populations, some researchers have raised the question whether an all-or-nothing ASD classification should be replaced by a multimodal symptom-based assessment of health care needs (68). Based on our findings, we would argue that, at least in children with a syndromic form of ASD, a symptom-based approach might be favorable. This would ensure that individuals who experience ASD symptoms are eligible for treatment of these symptoms regardless of their diagnostic status. However, further research is required to better understand the effect of developmental delay on social skills measured by ASD screening instruments. While an all-or-nothing diagnosis is still the standard, the differences between these instruments in our study highlight the importance of a multimodal assessment of ASD symptoms to diagnose ASD reliably in these children.

### Cross-Syndrome Comparisons of Symptom Severity Scores

In line with our hypothesis, and in accordance with the ASD classification prevalence, ASD symptom severity overall was highest in the FXS group and lowest in the NF1 group when we compared the main subscales of the ADOS and SRS. Also in line with our expectations, the scores of the TSC and AS groups mostly fell between the FXS and NF1 scores. The TSC group showed higher symptom severity compared to the AS and NF1 group for the ADOS social affect domain, and higher scores than the NF1 group on the restricted and repetitive behavior domain of the SRS. Based on the similarities in the underlying pathways of FXS and AS, and of TSC and NF1, we hypothesized that we might also find similarities in symptom presentation between these groups. Contrary to our expectations, the overall ASD severity scores of the TSC group seemed to be more similar to those of the FXS group whereas the AS group scores appeared more similar to those of the NF1 group.

### Cross-Syndrome Comparisons of Symptom Severity Scores in Individuals With an ASD Classification

By comparing the syndrome groups only including individuals with an instrument-based ASD classification we reduced the within-group variability and we were able to compare our syndrome groups to a non-syndromic ASD group. The severity scores of the syndrome groups differed from those of the non-syndromic ASD group on several domains. Especially on the SRS, the syndrome groups showed less ASD symptom severity compared to non-syndromic ASD in the social domain. On the restricted and repetitive behavior domains on the other hand, the nsASD severity scores were lower than those of the FXS group, and higher than those of the TSC and NF1 groups. Despite some slight differences, the results of the subgroup comparisons were broadly similar for the ADOS and the SRS. On the main scores of the ADOS and SRS, the scores that determine whether someone qualifies for an ASD classification, the syndrome groups seem to perform relatively well in terms of social interaction and communication compared to the non-syndromic ASD group. As we discussed earlier, the DSM-5 requires that social communication difficulties are more impaired than would be expected based on the developmental level of an individual. Our findings suggest that a combination of severe developmental delay and relatively good social communication skills may prevent individuals with these syndromes from receiving an ASD classification or even a clinical diagnosis, which influences their access to health care services.

### Cross-Syndrome Symptom Profile Analysis in Individuals With an ASD Classification

Our comparison of the ASD symptom profiles revealed several syndrome-specific strengths and challenges. While the FXS group scored highest overall, our results demonstrated a specific relative challenge for this group in the restricted interests and repetitive behavior domain. On the other end of the spectrum, the NF1 group had the least severe ASD symptoms overall, and had a specific relative strength in the restricted and repetitive behavior domain. The low severity scores in NF1 could cause ASD symptoms to be more easily overlooked in clinical practice in this group compared to the other syndrome groups. The TSC group also showed more challenges in the restricted and repetitive behavior domain, but their scores in this domain were lower than the FXS group and the nsASD group. For the AS group we found a relative strength on the social communication and social motivation domains, and a relative weakness in the reciprocal social interaction and in the creativity and play domains of the ADOS. Despite the fact that for all children with AS the lowest module was selected, this effect is likely due to a discrepancy between the developmental level of the AS group and the demands that some ADOS items put on the children. Therefore, in clinical practice, all scores that require a certain cognitive level must be interpreted cautiously for individuals with severe developmental delay.

While the genetic variations in FXS and AS, and in NF1 and TSC, respectively, affect neurodevelopmental pathways in a similar manner, the syndromes are fundamentally different in their origin as well as their phenotypes. Therefore, it would be highly unlikely that these syndromes would show an identical pattern of ASD symptoms on all subscales. Despite several differences, the syndrome pairs did show similarities on a majority of the ASD symptom subscales. To further explore the relationship between the observed similarities in the ASD profiles and the genetic pathways affected in these syndromes deep phenotyping studies are recommended that, for example, include (neural) biomarkers.

### Strengths and Limitations

A strength of our study is that our sample was relatively free of selection bias. All assessments were carried out in the context of regular clinical care and all children were assessed regardless of whether ASD was suspected. This was supported by the fact that we did not find a difference in ASD severity between the children with and without an IQ assessment. Nevertheless, a selection bias may still exist for the FXS group. As the somatic problems are often mild or absent in FXS—as opposed to NF1, TSC and AS—it is possible that only children with more severe (behavioral) problems choose to visit the ENCORE center of expertise.

Even though our sample is relatively large for a cross-disorder comparison between rare disorders, and the sample is relatively free of bias, we could not control for neurobiological variability within the syndrome groups (e.g., the deletion status in AS, the mutation type in TSC, the locations of neuronal tubers or abnormal tissue growth in NF1 and TSC, or the genetic mosaicism and gender differences related to the X-linked nature of FXS). We also did not account for other comorbidity within our sample, such as epilepsy, ADHD or anxiety disorders. While ASD, social anxiety and ADHD are distinct neurodevelopmental conditions, their symptoms do overlap and therefore comorbidity of ADHD and/or anxiety with ASD may influence scores on both the ADOS and the SRS. Especially in FXS, social anxiety is very common, in children with and without an ASD diagnosis (87). Future in-depth studies should explore these potentially contributing factors in these individual syndromes and study their effect on the development and presentation of ASD symptoms. In the subgroup analyses the sample sizes were even smaller as we included only individuals with an ADOS or SRS ASD classification. We chose to use an instrument-based classification for both the SRS and ADOS separately, instead of DSM-5 criteria, or a combined ADOS and SRS classification, for our subgroup selection because we wanted to examine the broad spectrum of ASD symptoms regardless of whether individuals qualified for a DSM-5 classification, or whether they qualified on both instruments. Although conclusions on the presence of clinical ASD diagnoses within these syndrome groups could not be drawn with this approach, it enabled us to examine both clinical and sub-clinical symptoms present in these syndromes. While a reduction of sample sizes in our subgroup analyses was expected, given the prevalence of ASD in these syndromes, the results of the subgroup analyses should be interpreted cautiously. Therefore, replication of this study in a larger sample, while accounting for syndrome specific features, would be necessary to validate the results of this study. This may be achieved by combining data from large natural history studies, such as the FORWARD study on FXS (88) and the TSC Natural History Database (TSC Alliance) for example. Data from the FORWARD study shows that 87% of children with FXS would classify as having ASD on the SRS, irrespective of ASD diagnosis (89), which supports the findings of this study. However, because ASD is not as prevalent in all syndromes, ASD screening is not standard practice for all syndromes, so selection bias should be accounted for.

Regarding gender differences, males in general are more likely to receive an ASD diagnosis than females (90). In addition, we know that FXS is more prevalent in males, and that males with FXS are more severely affected, in terms of developmental level as well as ASD symptomatology. Therefore, we added gender as a covariate in our analyses. The proportion of males vs. females in our FXS group was high at ~75%. Because the prevalence of ASD in males is higher than in females, in FXS (33) as well as in general (33, 91), including more males may have resulted in a higher ASD classification prevalence in our FXS sample compared to other studies, and a lower developmental level. Even though our sample size was relatively large for a cross-disorder comparison between rare disorders, the number of included females was not sufficient for stratification into gender subgroups. From studies in non-syndromic ASD as well as syndrome groups, we know that gender affects the pattern of ASD symptom presentation (90). Future studies should stratify their data in order to reveal the impact of gender on the presentation of ASD characteristics in males vs. females.

Another limitation of this study is the difference in developmental level between our groups, especially between the syndrome groups and the nsASD group. As our FXS group contained more males than expected, and the IQ of males with FXS is generally lower than that of females with FXS, the IQ of our FXS group may be relatively low compared to more balanced samples. It is known that the developmental level of children and young adults can influence the measurement of ASD symptom severity (77, 78). The ADOS allows clinicians to choose a module based on the developmental level of the participant, which is estimated based on the level of spoken language. However, the cognitive profiles of syndrome groups also show syndrome specific strengths and challenges. Individuals with similar levels of spoken language may vary greatly in other areas of cognitive and motor development. There are no specific norms available for the ADOS for samples with developmental delay or complex behavioral problems. In our study we compared symptom severity in the syndrome groups to a non-syndromic ASD sample in order to better interpret the group differences that we have found. However, it is important to keep in mind that the developmental level of this non-syndromic ASD sample was higher than that of our syndrome groups. We have attempted to reduce the effect of developmental levels on our results by adding IQ or DQ scores as a covariate in our analyses, but as IQ/DQ levels are likely to vary within and between our groups, our results should be interpreted cautiously.

## Conclusion

The syndrome-specific strengths and challenges we found in FXS, TSC, AS, and NF1 provide a frame of reference to evaluate an individual's symptom severity relative to the syndromic population as a whole and to guide treatment decisions. Based on the overall ASD symptom profile, clinicians should closely monitor the development of ASD-symptoms, taking into account the syndrome-specific strengths and weaknesses within the ASD profile when selecting treatment methods. Similarities in ASD symptom profiles between AS and FXS, and between NF1 and TSC may be caused by similarities in their underlying neurobiological pathways. Deep phenotyping studies are required to link symptom patterns to specific neurobiological pathways more directly. Additionally, the variation in symptom severity within our sample also highlights the need to investigate patterns of ASD symptom severity within syndromes, which might reveal subgroups with a more homogeneous symptom presentation.

## Encore Expertise Group

Rianne Oostenbrink^1, 2, 3^, Karen C.G.B. Bindels-de Heus^1, 3^, Marie-Claire Y. de Wit^1, 4^, Henriëtte A. Mol^1, 3^ and Ype Elgersma^1, 5^.

^1^ENCORE Expertise Center for Neurodevelopmental Disorders, Erasmus MC University Medical Center, Rotterdam, Netherlands

^2^Full Member of the European Reference Network on Genetic Tumor Risk Syndromes (ERN GENTURIS)—Project ID No 739547

^3^Department of General Pediatrics, Erasmus MC, Rotterdam, Netherlands

^4^Department of Neurology, Erasmus MC, Rotterdam, Netherlands

^5^Department of Clinical Genetics, Erasmus MC, Rotterdam, Netherlands

## Data Availability Statement

The original contributions presented in the study are included in the article/Supplementary Materials, further inquiries can be directed to the corresponding author/s.

## Ethics Statement

The studies involving human participants were reviewed and approved by Medical Ethics Committee of the Erasmus University Medical Center, Rotterdam, Netherlands (MEC-2015-203 and MEC-2011-078). Written informed consent to participate in this study was provided by the participants' legal guardian/next of kin.

## Author Contributions

BD, LH, PN, JL, AR, the ENCORE Expertise Group, SM, and GD were involved in the design of the ENCORE database and supervised data collection. ES, KL, and DH gathered and prepared the dataset. KG-L provided the data of the Social Spectrum Study. KL performed the analyses. SM, GD, and MH were involved in the planning and supervision of the project. KL and ES prepared the manuscript in consultation with SM, BD, and GD. All authors discussed the results and contributed to the final manuscript.

## Funding

This research was financially supported by the Sophia Children's Hospital Fund (Rotterdam, Netherlands) under grant numbers S16-14 and B14-02. This research was supported (not financially) by the European Reference Network on Genetic Tumor Risk Syndromes (ERN GENTURIS) – Project ID No 739547. ERN GENTURIS is partly co-funded by the European Union within the framework of the Third Health Programme ERN-2016-Framework Partnership Agreement 2017–2021.

## Conflict of Interest

The authors declare that the research was conducted in the absence of any commercial or financial relationships that could be construed as a potential conflict of interest.

## Publisher's Note

All claims expressed in this article are solely those of the authors and do not necessarily represent those of their affiliated organizations, or those of the publisher, the editors and the reviewers. Any product that may be evaluated in this article, or claim that may be made by its manufacturer, is not guaranteed or endorsed by the publisher.
