# Supplementary figures and images for: Autism Symptoms in Children and Young Adults With Fragile X Syndrome, Angelman Syndrome, Tuberous Sclerosis Complex, and Neurofibromatosis Type 1: A Cross-Syndrome Comparison
Source: Front Psychiatry. 2022 May 16;13:852208. doi: 10.3389/fpsyt.2022.852208 (PMC9149157; doi:10.3389/fpsyt.2022.852208)

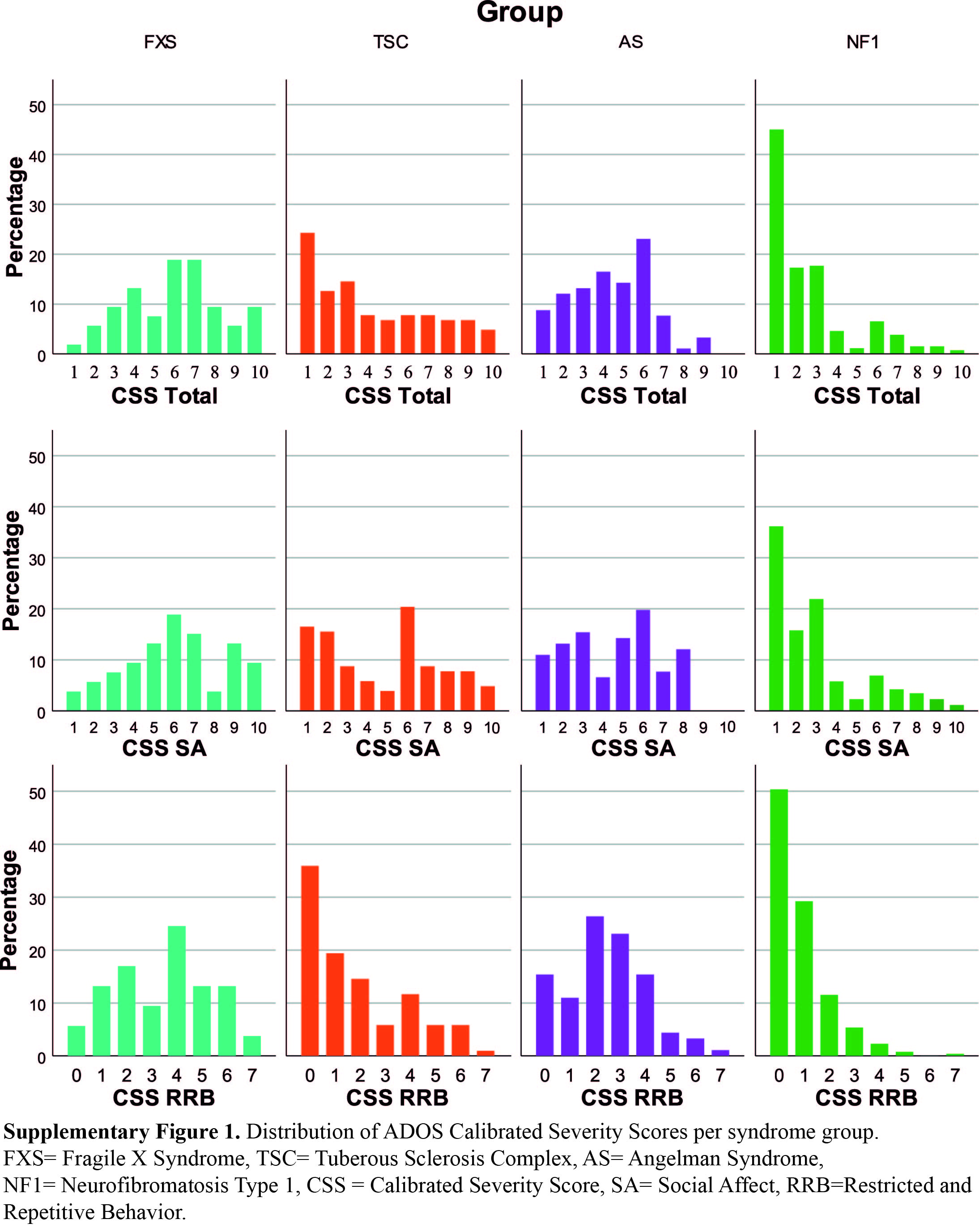

Supplement: Supplementary file 1 [file Image_1.JPEG]

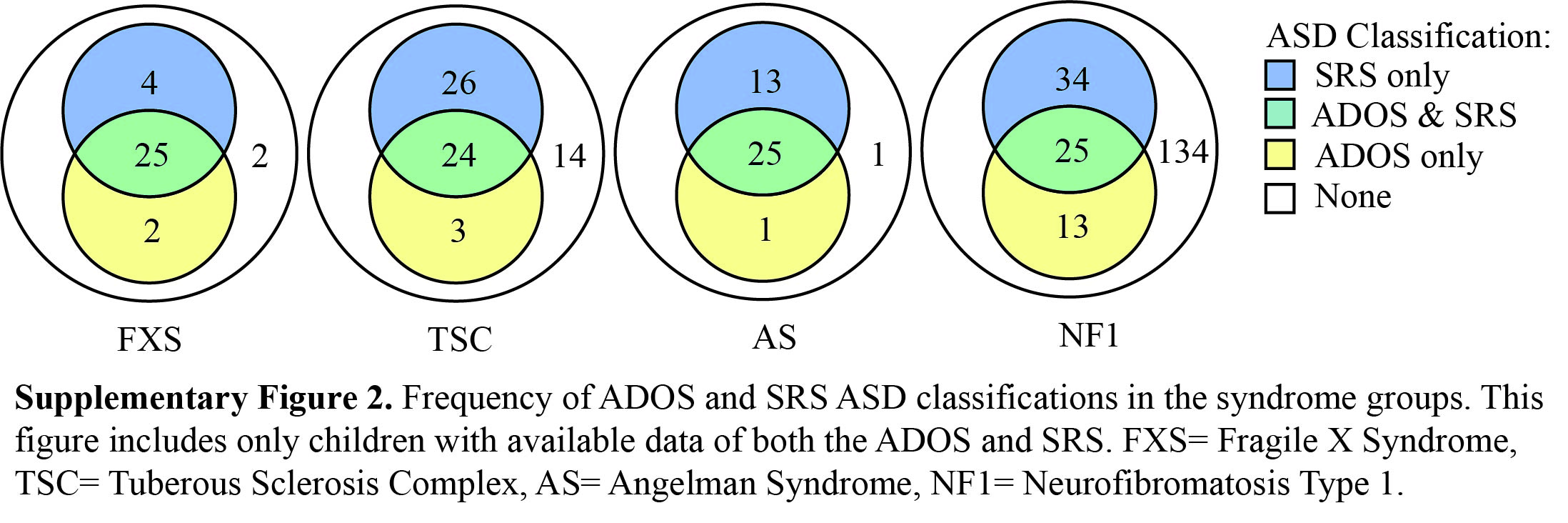

Supplement: Supplementary file 2 [file Image_2.JPEG]
