# Supplementary material for: Autism Symptoms in Children and Young Adults With Fragile X Syndrome, Angelman Syndrome, Tuberous Sclerosis Complex, and Neurofibromatosis Type 1: A Cross-Syndrome Comparison
Source: Front Psychiatry. 2022 May 16;13:852208. doi: 10.3389/fpsyt.2022.852208 (PMC9149157; doi:10.3389/fpsyt.2022.852208)
Supplement: Supplementary file 3 [file Table_1.DOCX]

**Supplementary Table 1.** Cross-syndrome comparison of the ASD symptom severity profiles

| Dependent Variable | *df* | *df* error | F | η^2^ | FXS | TSC | AS | NF1 | nsASD |
| --- | --- | --- | --- | --- | --- | --- | --- | --- | --- |
|  |  |  |  |  | M(SD) | M(SD) | M(SD) | M(SD) | M(SD) |
|  | ADOS WSS – ASD subsample | | | | | | | | |
| Multivariate | 16 | 804.1 | 4.270** | .060 | *Wilks’ Λ=.779* | | | | |
| WSS_LCOM_ | 4 | 266 | 2.175 | .032 | .397 (.162) | .362  (.140) | .327  (.114) | .316  (.143) | .319  (.142) |
| WSS_RSI_ | 4 | 266 | .098 | .001 | .535  (.190) | .530  (.237) | .595  (.185) | .453  (.141) | .453  (.142) |
| WSS_CREA_ | 4 | 266 | 8.504** | .113 | .385  (.371)_c_ | .581  (.396) | .875  (.208)_ab_ | .412  (.378)_cd_ | .524  (.338)_bd_ |
| WSS_RRB_ | 4 | 266 | 4.132** | .059 | .389  (.206)_a_ | .308  (.255) | .354  (.224) | .132  (.157)_b_ | .121  (.130)_b_ |
|  | SRS T-scores subscales - ASD subsample | | | | | | | | |
| Multivariate | 20 | 1155 | 3.646** | .049 | *Wilks’ Λ=.816* | | | | |
| T_SAw_ | 4 | 352 | 4.713** | .051 | 69.9  (12.3) | 66.5  (12.8)_a_ | 72.8  (9.8) | 64.7  (9.3)_a_ | 69.3  (10.1)_b_ |
| T_SCog_ | 4 | 352 | 4797** | 052 | 78.2  (10.9)_a_ | 77.3  (10.4)_a_ | 75.8  (6.2)_b_ | 73.1  (10.7) | 75.1  (10.6)_a_ |
| T_SCom_ | 4 | 352 | 5.723** | .061 | 79.2  (11.5)_ac_ | 75.6  (11.5) | 75.0  (8.9)_b_ | 72.0  (9.3)_ab_ | 76.8  (9.3)_c_ |
| T_SM_ | 4 | 352 | 9.198** | .095 | 76.5  (12.9)_a_ | 70.8  (13.4)_ac_ | 60.9  (10.0)_b_ | 66.4  (8.8)_bc_ | 72.7  (11.3)_a_ |
| T_AM_ | 4 | 352 | 6.438** | .068 | 87.8  (16.0)_a_ | 78.5  (15.0)_b_ | 83.6  (10.5)_bc_ | 73.4  (12.6)_b_ | 78.7  (12.8)_ac_ |
| *Note.* The means represent the uncorrected mean scores of the groups. For syndrome groups with the same subscript letter the mean CSS or subscale score is not significantly different at the *p*=0.05 level (Bonferroni corrected). No subscript letter means the group does not differ from any other group. ASD = Autism Spectrum Disorder, FXS= Fragile X Syndrome, TSC= Tuberous Sclerosis Complex, AS= Angelman Syndrome, NF1= Neurofibromatosis Type 1, nsASD= non-syndromic ASD, WSS = Weighted subscale score, LCOM= Language and communication, RSI= Reciprocal Social Interaction, CREA = Creativity and Play subscale, RRB = Restricted and Repetitive Behavior, SAw = Social Awareness, SCog= Social Cognition, SCom= Social Communication, SM= Social Motivation, AM= Autistic mannerisms.. **p*<.05, ***p*<.01. | | | | | | | | | |
